# C-reactive protein levels: a prognostic marker for patients with head and neck cancer?

**DOI:** 10.1186/1758-3284-2-21

**Published:** 2010-08-02

**Authors:** Astrid L Kruse, Heinz T Luebbers, Klaus W Grätz

**Affiliations:** 1 Department of Craniomaxillofacial and Oral Surgery, University Hospital Zurich, Zurich, Switzerland

## Abstract

**Background:**

Recent advances in understanding complex tumor interactions have led to the discovery of an association between inflammation and cancer, in particular for colon and lung cancer, but only a very few have dealt with oral cancer. Therefore, the aim of the current study was to investigate the significance of preoperative C-reactive protein (CRP) levels as a parameter for development of lymph node metastases or recurrence.

**Materials and methods:**

In 278 patients with oral cancer, preoperative CRP levels were compared with development of recurrence and metastasis.

**Results:**

In 27 patients from the normal CRP group, and in 21 patients from the elevated CRP group, local recurrence was observed. Concerning lymph node metastases, 37 patients were in the normal group and 9 patients in the elevated CRP group. No significant correlation could be found between elevated CRP levels and metastasis (p = 0.468) or recurrence (p = 0.137).

**Conclusion:**

Our findings do not appear to support a correlation between preoperative CRP levels and development of recurrence or metastases. In further studies, CRP levels in precancerous lesions and in Human Papilloma Virus (HPV) positive patients with oral squamous cell carcinoma (SCC) should be studied.

## Introduction

In 1863, Rudolf Virchow postulated the induction hypothesis that cancer originated at site of chronic inflammation [1]. Chronic inflammation is associated with the risk of cancer. For instance, human immunodeficiency virus, viral hepatitis B, and human papilloma virus are well known for their association with an increased risk of cancer [1]. An argument for the hypothesis that inflammation is associated with cancer is also derived from the reduced risk for colorectal cancer that has been associated with long-term use of aspirin and other nonsteroidal anti-inflammatory drugs [2].

C-reactive protein (CRP) is an acute-phase protein and a marker for inflammation. The synthesis of CRP in the hepatocytes may be regulated by pro-inflammatory cytokines like interleukin-1, interleukin-6, and tumor-necrosis factor, which are also reported for different malignancies. Therefore, these pro-inflammatory cytokines are currently the subject of intense studies as influencing factors in various types of tumors. It is increasingly recognized that in addition to tumor stage, the disease progression depends on a complex interaction between the tumor and the host's inflammatory response.

Two hypotheses could be associated with increased CRP levels as a sign of chronic inflammation. First, the induction hypothesis: chronic inflammation results in excessive cell proliferation and activation of a cascade of cellular actions, leading to induction of irreversible DNA damage [1]. Second, the response hypothesis: the immune response of the host as a consequence of tumor growth itself could be the reason for the elevation in CRP levels [3]. However, it is still unclear whether CRP levels are elevated before the biological onset of cancer or if an elevated CRP level is also a risk factor for the development of cancer.

Findings from the studies, however, have been inconsistent. Some authors have observed an association between elevated serum CRP levels in some cancers, like colorectal [4-6] and lung [2]. On the other hand, some researchers doubt that CRP can be regarded as a prognostic marker [7]. However, raised CRP concentrations have been demonstrated to be an indicator of a poorer prognosis for squamous cell carcinoma (SCC) in patients with esophageal cancer [8,9], but concerning the cancer of the oral cavity only a very few studies have dealt with this topic so far (Table 1). All of these data are consistent with the hypothesis that CRP levels increase after onset of oral cancer.

**Table 1:** Studies dealing with the association between oral SCC and preoperative CRP levels

Author	Number of patients	Results
Gallo et al. [10]	18	Significance of CRP and IL-6 in regard to tumour stage

Jablonska et al. [11]	42	CRP, IL-1β, IL-6, TNF-α serum levels related to clinical stage of disease

Khandavilli et al. [12]	60	CRP level is associated with worse overall outcome

Therefore, the aim of the current study was to investigate the significance of preoperative CRP levels as a parameter for development of lymph node metastases or recurrence.

## Materials and methods

Chosen for evaluation were 278 patients (119 female, 159 male) with oral SCC that were treated between 1999 and 2008 at a single center (Department of Craniomaxillofacial and Oral Surgery, University Hospital Zurich). All serum CRP levels (obtained between one and 5 days prior to surgical treatment), recurrence rate, and lymph node metastases were taken into consideration. The minimum follow-up time was 12 months. Exclusion criteria were inadequate information and a follow-up time of less than 12 months.

The patients were divided into two groups according to the preoperative measure of C-reactive protein concentration: those with CRP values in the normal range (< = 5.0 mg/L), and those with elevated RP levels (>5.0 mg/L) according to Khandavilli et al. [12] and Komai et al. [13].

For statistical analysis SPPS 18 (SPSS Inc, Chicago, IL) for the Mac was used. P value < 0.05 was considered to be statistically significant. Kaplan-Meier analysis with log-rank testing was used for univariate analysis.

## Results

**Figure 1:**
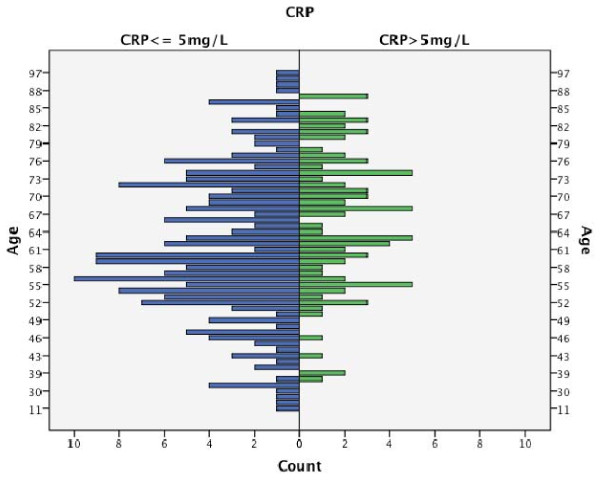
**Distribution of age and CRP**.

Out of 278 patients with a mean CRP of 7.36 mg/L, 193 (69.4%) patients had a preoperative CRP level < = 5 mg/L; 85 (30.6%) patients had a CRP level >5 mg/L; and the distribution was independent of age (Fig. 1).

Local recurrence was seen in 48 patients (17.3%), with a mean time of 24.31 months (range: 7-84 months); 2cd tumors in 24 (8.6%); and no recurrence in 206 (74.1%). Cervical lymph node metastases were observed in 46 patients (16.5%) after a mean time of 18.27 months (range: 4-71 months), distant metastases in 14 (5%), and no metastases in 218 patients (78.5%).

**Figure 2:**
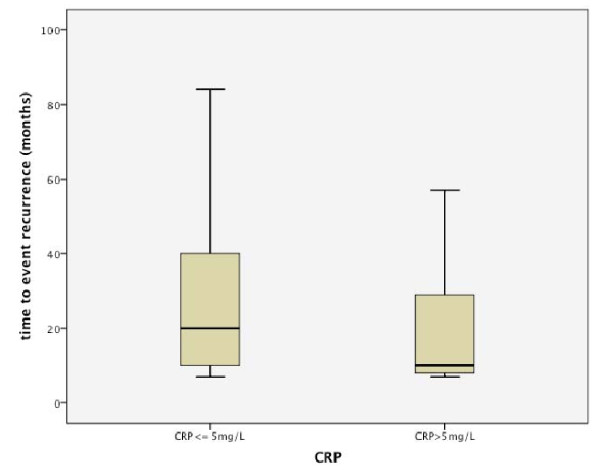
**Distribution of time to recurrence dependent on CRP level**.

**Figure 3:**
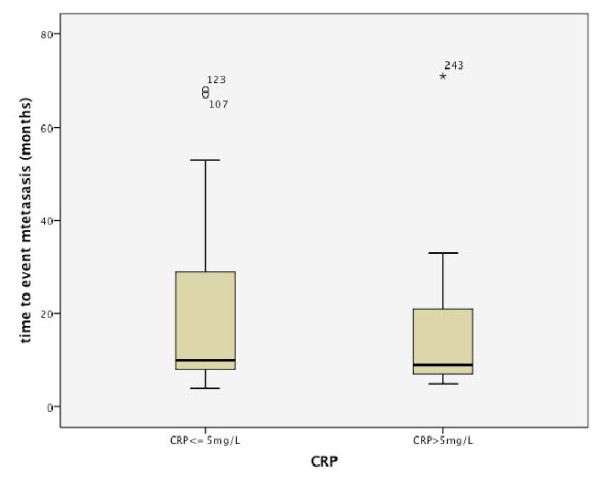
**Distribution of time to metastases dependent on CRP level**.

It was striking that although recurrence appeared earlier in the elevated CRP group (Fig. 2), no difference was found concerning the time of metastases (Fig. 3).

**Figure 4:**
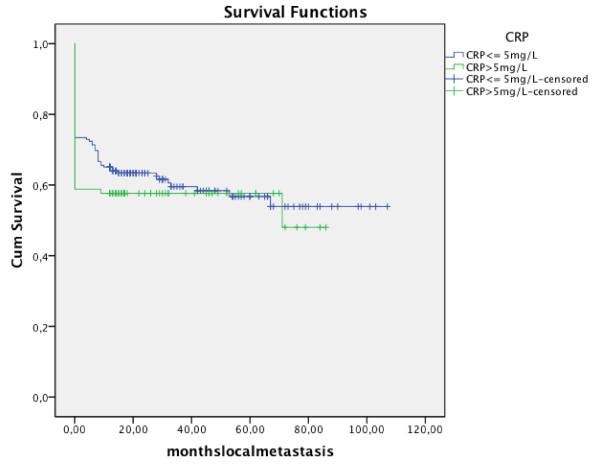
**Kaplan-Meier analysis of development of metastasis in regard to CRP level**.

**Figure 5:**
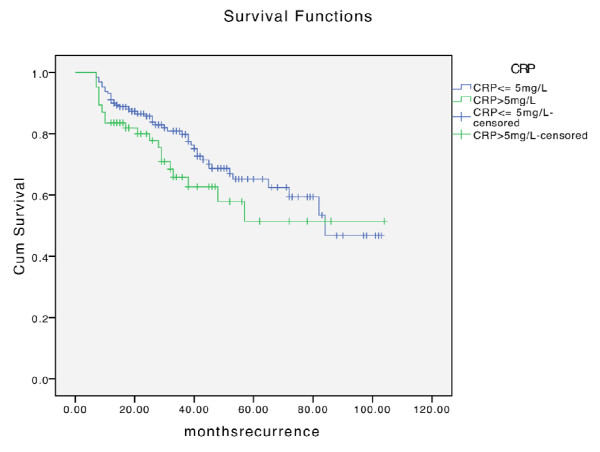
**Kaplan-Meier analysis of development of recurrence in regard to CRP level**.

In 27 patients in the normal CRP group and in 21 patients belonging to the elevated CRP group, local recurrence was observed. Concerning lymph node metastases, 37 patients were in the normal group and 9 patients in the elevated CRP. No significant correlation was found neither for development of metastasis (p = 0.468) (Fig. 4) nor for recurrence (p = 0.137) (Fig. 5).

## Discussion

In order to establish prognostic predictors for oral SCC, several studies have been performed. The purpose of our study was to find a simple and cost effective indicator for oral SCC. The mean follow-up time was 35.97 months (range: 12-107 months).

In this study, the mean value of serum CRP was 7.36 mg/L; the serum level was raised in 85 patients (30.6%); the increase was mostly moderate (Fig. 1); and a high increase of more than 50 mg/L, seen in infectious disease, was found in 6 patients. The proportion of lymph node metastasis in the group of elevated CRP levels was smaller than that in the patients without CRP elevation. But no significant association between raised CRP levels and development of recurrence or metastases could be seen. Therefore this study does not confirm the results from other studies (Table 1) and we also disagree with Zingg U et al. [14] who suggested CRP-measurements in the re-staging process in patients who have undergone neoadjuvant treatment for esophageal cancer in order to help to select patients who are likely to benefit from surgery.

In the literature there seems to be inconsistency concerning the CRP levels: some authors have described CRP levels of more than 5 mg/L as raised (13, 14), while others have considered a more precise differentiation into three groups: low (<1 mg/L), average (1.0 - 3.0 mg/L), and high (>3.0 mg/L). In the current study, a CRP level of more than 5 mg/L was considered as raised. But one has to keep in mind that CRP levels can be reduced with smoking cessation [15] and weight loss[16,17].

Patients with cancer of the oral cavity can be in poor nutritional condition. For esophageal cancer, a correlation has been shown between elevated serum CRP concentration and malnutrition with impaired immunity [9]. Furthermore, smoking and alcohol abuse can also lead to chronic inflammation in the oral mucosa. Therefore, it would be of interest to investigate CRP levels in precancerous lesions--e. g., erosive lichen. Ki et al. [18] reported a significant correlation between the presence of acute mucositis and CRP level in 40 patients during radiotherapy for primary laryngo-pharyngeal cancer.

A strength of the current study was the high number of patients. One limitation is that CRP was measured at one point in time. Therefore intraindividual variations were not considered. Furthermore, general diseases associated with possible higher inflammation markers like diabetes mellitus or Morbus Crohn were not taken into consideration due to lack of informations.

However, CRP is a nonspecific marker of inflammation, and additional studies of specific cytokines that regulate acute-phase response are necessary to elucidate the mechanisms by which inflammation influences the risk of cancer.

## Conclusion

In summary, our findings do not appear to support a positive association between preoperative CRP levels and oral SCC. Further studies should examine CRP levels in precancerous lesions and in HPV positive patients with oral SCC.

## Competing interests

The authors declare that they have no competing interests.

## Authors' contributions

AK drafted the manuscript. TL participated in the design of the study. KG participated in its design and coordination. All authors read and approved the final manuscript.
